# Single Nucleotide Polymorphisms of FCRL3 in Iranian Patients with Behcet’s Disease

**Published:** 2019-06

**Authors:** Farhad SHAHRAM, Javad KAZEMI, Mahmoud MAHMOUDI, Zohreh JADALI

**Affiliations:** 1. Rheumatology Research Center, Shariati Hospital, Tehran University of Medical Sciences, Tehran, Iran; 2. Department of Clinical Genetics, National Institute of Genetic Engineering and Biotechnology, Tehran, Iran; 3. Department of Biostatics and Epidemiology, School of Public Health, Tehran University of Medical Sciences, Tehran, Iran; 4. Department of Immunology, School of Public Health, Tehran University of Medical Sciences, Tehran, Iran

**Keywords:** Behcet disease, Fc receptor like 3 (FCRL3), Polymorphism, Autoimmunity

## Abstract

**Background:**

Both genetic and environmental factors influence, susceptibility to autoimmune disorders including Behcet’s disease (BD). FCRL3 (Fc receptor like 3 genes), a novel immunoregulatory gene, has recently been reported as a new promising candidate gene for general autoimmunity. This study was conducted to explore the potential association of FCRL3 polymorphisms with BD.

**Methods:**

This study was conducted from 2010 to 2015 in Tehran University of Medical Sciences, Tehran, Iran. Four single-nucleotide polymorphisms of FCRL3 (rs7528684, rs11264799, rs945635, and rs3761959) were genotyped in 220 patients and 220 healthy controls. Typing of the polymorphisms in this case-control study was carried out using polymerase chain reaction-restriction fragment length polymorphism analysis.

**Results:**

Analysis of the alleles revealed a significantly lower frequency of the A allele at the −169 site (rs7528684) in BD patients compared with that in controls (*P*=0.000, 66.4% versus 82%, χ^2^= 30.23). Moreover, a significant lower frequency of AA genotype and higher frequency of GG genotype was recorded for rs7528684. There was also relationship between posterior uveitis as a clinical sign of disease and polymorphism of allele A at the −169 site (*P*=0.015).

**Conclusion:**

This study revealed a significant difference in both allele and genotype frequency at position −169 of FCRL3 gene between Iranian patients with BD and normal subjects. These data suggest FCRL3 gene polymorphisms might be the autoimmunity risk factor for BD.

## Introduction

Behçet’s disease (BD) is a chronic relapsing multisystemic disorder characterized by mouth and genital ulcerations, defined skin lesions, and uveitis (1). Although the etiology of disease remains unclear, many studies convincingly confirm the involvement of the immune system in disease pathogenesis. There is also growing evidence that supports the role of genetic factors in predisposition toward BD and a number of these susceptibility genes may lie within immune-related genes (1, 2). Single nucleotide polymorphism (SNP) genotyping method is an invaluable tool for association studies. This technique makes it possible to calculate the relevance between genetic variants and the presence/absence of a disease or a certain phenotype. The widespread occurrence of polymorphism among genes of the immune system also highlights the importance of this method for a better understanding of the molecular mechanisms responsible for pathogenesis of autoimmune diseases such as BD (2).

Several polymorphisms (including HLA-B51, TNF-α, GTPases of immunity associated protein(GIMAP), IL1A-IL1B, IRF8, and CEBPBPTPN1 have been reported in immune-related genes of patients with BD associated with disease susceptibility (3–6).

In recent years special attention is paid to studies concerning the FCRL3 gene polymorphisms (7). FCRL3 is a cell surface protein homologous to Fc receptors (FcRs) that in humans is encoded by the FCRL3 gene. This gene is a member of a recently identified FCRL gene family cluster on human chromosome 1q21–23 adjacent to the FcR genes (8). FCRL3 is expressed predominantly on B cells and its cytoplasmic tail is equipped with activating (ITAM) and inhibitory (ITIM) elements. Both motifs are evolutionarily conserved signaling module that duels for control of cellular activation in the immune system. This unique feature confers a dual positive and negative regulatory role on FCRL3 during B-cell antigen receptor signaling (9). The analysis of FCRL3 polymorphisms seems important because the results of different studies demonstrate an association between functional variant in FCRL3 and increased risk of autoimmunity including rheumatoid arthritis (RA), multiple sclerosis, autoimmune thyroid diseases (AITDs) and allergic rhinitis (10–13).

Others studies have also identified a strong relationship between polymorphisms of FCRL3 and BD (14). Nonetheless, studies to replicate these data have not been undertaken. Therefore, the aim of this study was to assess the influence of FCRL3 polymorphisms on the risk of BD among Iranian population.

## Materials and Methods

In this case-control study, venous blood was drawn from 220 patients with BD (137 men, 83 women) in BD outpatient clinic, Rheumatology Research Center, Shariati Hospital, at the Tehran University of Medical Sciences, Tehran, Iran, in 2010–2015. Inclusion criterion was fulfillment of the classification criteria of the revised International Criteria for BD (15). Moreover, there was no age limit or gender restriction. The mean age of the patients in this study was 38.37 ± 0.70 yr (men 37.01 ± 0.77 yr; women 40.61 ± 1.32 yr). The mean duration of BD was 14.66±0.57 yr (women 16.69 ± 1.03 yr; men 13.32 ± 0.65).

For controls, peripheral blood samples were collected from 220 healthy volunteers (163 men, 57 women) referred to Iranian Blood Transfusion Organization, Tehran, Iran. All controls were negative for BD, other rheumatologic or autoimmune diseases and oral aphthosis and their mean ages were 39.36 ± 0.73 yr (men, 40.36 ± 0.82 yr, women, 36.28 ± 1.49 yr).

Patients display a diverse spectrum of symptoms, affecting different organ systems. The clinical and demographic characteristics of the 220 BD patients are summarized in Table 1. Pseudo folliculitis and erythema nodusum were the main cutaneous lesions found in patients with BD. Other skin manifestations include papulopustular lesions and acneiform nodules. Ophthalmologic manifestations, comprising anterior uveitis, posterior uveitis, and retinal vaasculitis. Joint manifestations refer to arthralgia, arthritis, and anky-losing spondylitis; neurological complications consisted of central and peripheral manifestations; vascular involvement of patients was in the form of arterial thrombosis, phlebitis, superficial phlebitis, and large vein thrombosis. Other symptoms appeared within gastrointestinal tract, heart, and kidney.

**Table 1:** Clinical and demographic features of the BD patients and controls

***Features***	***Patients***	***Controls***
Number	220	220
Age(yr)	38.37±0.70	39.36±0.73
Duration of disease(years)	14.66±0.57	
Gender(Female/Male)	83/137	57/163
Oral aphthosis, n/total n (%)	218/220(99.1)	
Genital aphthosis, n/total n (%)	133/220(60.5)	
Skin lesions, n/total n (%)	128/220(58.2)	
Ophthalmic manifestations, n/total n (%)	152/220(69.1)	
Joint manifestations, n/total n (%)	113/220(51.4)	
Neurological involvement, n/total n (%)	25/220(11.4)	
Vascular involvement, n/total n (%)	21/220(9.5)	
Other manifestations, n/total n (%)	22/220(10)	
Pathergy phenomenon	94/220(42.7)	
Family history of BD	10/220(4.5)	

n/total n = number of individuals/total number of patients or controls analyzed; BD=Behcet’s disease

### Ethics statement

The protocol was approved by the Research Ethics Board at Tehran University of Medical Sciences. All participants had completed the informed consent prior to start of any study-related procedures.

### DNA extraction

Blood samples collected in evacuated glass tubes containing EDTA and DNA was extracted from whole blood using the DNA extraction kit (MBST, Iran).

The quality and quantity of DNA were estimated by agarose gel electrophoresis and spectrophotometry. DNA samples were stored in −20 ºC until the day of assay.

### PCR amplification

Genotype determination was carried out through polymerase chain reaction (PCR) with subsequent restriction fragment length polymorphism (RFLP) analysis. Four SNPs spanning the FCRL3 gene were used in our association studies, −169A/G (rs7528684), −110C/T (rs11264799), +358C/G (rs945635), and +1381 A/G (rs3761959). PCR primers pairs were designed by using OLIGO software (National Biosciences) and purchased from Macrogen (Seoul, Korea). The specific sequences of primers and enzymes used in this study are shown in Table 2. PCR reaction was performed with 1.5μl of 10× reaction buffer, 0.5 μl of 50 mM MgCl_2_ (Cinna Gen Inc. Tehran, Iran), 0.5μl of 10 mM dNTPs (Cinna Gen Inc. Tehran, Iran), 0.5 μl of forward and reverse primers (100 pmol/μl) for each gen stated in Table 2, 0.1 μl of 5U/μl Taq DNA polymerase (Fermentase; Glen Burnie, Maryland, USA) and 1.2 μl extracted DNA (10 ng/μl) as template. The total volume of the PCR reaction mixtures was adjusted to 25 μl with distilled deionized water.

**Table 2:** Primer sequences and restriction enzymes used in studied polymorphisms

***SNPs***	***dbSNP ID***	***Primer sequences***	***Restriction enzyme***
−169A/G(fcrl3_3)	rs7528684	F:5′GAAAATAATACAAATGTACAGATTA3′ R:5′GGCTTTAAAAACGGTGGTAC3′	BsmFI
−110C/T(fcrl3_4)	rs11264799	F:5′CTCAATCCCGGTAGTGATACA3′ R:5′CTCATAAACAACTTATGTGAGA3′	Ple 1
+358C/G(fcrl3_5)	rs945635	F:5′TTATAGCCCATCTACTCACTCAGGATCA3′ R:5′CCGGGATTGAGATACAAACAGCATTT3′	HaeIII
+1381A/G(fcrl3_6)	rs3761959	F:5′TCCGACTTTTTCAGTCTCTAGGTTTT3′ R:5′TGATAGCAGCACTAGCTTGGACATTCA3′	MspI

SNP, single nucleotide polymorphisms; dbSNP, database of single nucleotide polymorphism; F, forward primer; R, reverse primer

The thermal cycling conditions for PCR reactions were: initial denaturation at 95 °C for 5 min, followed by 28–35 cycles of denaturation at 95 °C for 50 sec; primer annealing for 56 sec at 58 °C (rs7528684), 50 sec at 57 °C (rs11264799), 63.5 °C (rs945635), 59 °C (rs3761959) and 50 sec extension at 72 °C. All PCR products were subjected to a final extension step for 10 min at 72 °C. In the next step, PCR amplicons were electrophoresed on 1% agarose gel and visualized by DNA Safe Stain (CinnaGen, Tehran, Iran).

### Restriction Fragment Length Polymorphism (RFLP)

The amplified products were digested overnight by addition of position specific restriction enzymes (Fermentas, Canada). Buffers and digestion temperatures were applied according to the manufacturer’s protocols (CinnaGen, Tehran, Iran). The digested DNA fragments were submitted to 8% polyacrylamide gel electrophoresis and stained with 0.1% silver nitrate (Merck, Germany). The genotypes of each polymorphism were determined according to the digestion patterns. To confirm the results of genotyping by the PCR-RFLP, 10% of random samples were selected to perform a direct sequencing analysis.

### Statistical analysis

Deviation from the Hardy-Weinberg equilibrium for all SNPs was calculated in both cases and controls using the chi-square (χ^2^) test. The frequency of genotypes and alleles were determined using the standard Chi-square (χ^2^) and Fisher exact tests. A *P*-value<0.05 was considered statistically significant.

The odds ratio (OR) and associated 95% confidence intervals (95% CI) for patients versus controls were also calculated by conditional multivariate logistic regression analysis.

## Results

Overall, 220 BD patients and 220 healthy controls were genotyped for the rs7528684, rs11264799, rs945635, and rs3761959 SNPs. The allele and genotype frequencies for these SNPs are indicated in Table 3. All the cases and controls were in Hardy-Weinberg equilibrium (*P*>0.05). There were significant differences between the BD patients (n=220) and controls (n=217) concerning the frequencies of rs7528684. A significantly decreased prevalence of the rs7528684 AA genotype and A allele was found in the BD patients compared to the controls (*P*=0.000, OR [95% CI]=0.43[0.32–0.59]; *P*=0.000, OR [95% CI]=2.19 [1.49–3.22], respectively. In contrast, the frequency of the GG genotype in patients was significantly higher than that in controls [*P*=0.000, OR [95% CI]=0.089[0.027–0.296]. Moreover, there was no significant difference between the AG genotype in two groups [*P*=0.08, OR [95% CI]=0.75 [0.50–1.10].

**Table 3:** Frequencies of alleles and genotypes of four FCRL3 SNPs in BD patients and controls

***SNPs***	***Alleles/genotypes***	***BD n = 220(%)***	***Controls n = 220(%)***	***χ^2^***	***P***	***OR (95% CI)***
rs7528684	AA	102(46.4)	142(65.4)	30.23	0.01	1.70(1.14–2.54)
	AG	88(40)	72(33.2)		0.001	0.12(0.04–0.42)
	GG	30(13.6)	3(1.4)			
Alleles	A	292(66.4)	356(82)	27.96	0.000	0.43(0.32–0.59)
	G	148(33.6)	78(18)			
rs11264799	AA	80(36.4)	75(34.4)	0.60	0.54	0.88(0.58–1.33)
	AG	101(45.9)	108(49.5)		0.52	0.84(0.49–1.43)
	GG	39(17.7)	35(16.1)			
Alleles	A	261(59.3)	258(59.2)	0.002	1.00	1.01(0.77–1.32)
	G	179(40.7)	178(40.8)			
rs945635	CC	71(32.3)	86(39.1)	2.64	0.21	1.30(0.86–1.95)
	CG	119(54.1)	111(50.5)		0.52	0.82(0.45–1.50)
	GG	30(13.6)	23(10.5)			
Alleles	C	261(59.3)	283(64.3)	2.33	0.15	0.81(0.62–1.06
	G	179(40.7 )	157(35.7)			
rs3761959	AA	31(14.1)	34(15.5)	1.05	0.91	1.03(0.60–1.79)
	AG	113(51.4)	120(54.5)		0.35	0.82(0.54–1.24)
	GG	76(34.5)	66(30)			
Alleles	A	175(39.8)	188(42.7)	0.79	0.41	0.89(0.68–1.16)
	G	265(60.2)	252(57.3)			

BD = Behcet disease; OD=odds ratio; CI=95% confidence interval

No statistically significant difference was observed regarding the alleles and genotypes of the other three SNPs between patients with BD and normal subjects (Table 3). In another part of this study, the relationship between the SNPs and different clinical manifestations was analyzed. A significant association existed between allele A in −169 site and posterior uveitis (*P*=0.02, OR [95% CI]=0.48 [0.27–0.86]). Of the 220 patients 83(37.73%) were women and 137(62.27%) were men. The male to female ratios of cases were 1.65 (137: 83) and no statistically significant difference was observed in the SNPs between male and female patients.

## Discussion

In the current study, the association of four FCRL3 SNPs with BD were analyzed in Iranian population. The frequency of the FCRL3 gene polymorphism at position −169 showed a significantly lower frequency of the AA genotype and the allele A in BD patients compared to healthy controls (*P*<0.05). In contrast, a significant increase in frequency of GG genotype was observed.

These findings are consistent with earlier results, with emphasis on the association between −169 A/G polymorphism and autoimmune diseases*.* For instance, evidence were provided regarding a significant increase in the prevalence of the AA genotype and A allele of rs7528684 in allergic rhinitis (12). In another study, approximately 1.4-fold found significantly raised frequency of the FCRL3_3 C allele (OR=1.41 [95% CI=1.08–1.84], *P*=0.013) in women with endometriosis-related infertility (16). Moreover, mutations of FCRL3_3, FCRL3_5, and 3_6 could increase the chances of Graves’ disease (13). Interestingly, patients with intractable graves’ disease had significantly lower frequency of the FCRL3 -169TT genotype than patients in remission period (17). Results of these surveys highlight the importance of FCRL3 polymorphisms in the pathogenesis of autoimmune diseases. However, the exact functions of these genes and the mechanisms for this genetic association is still unknown. Genetic variation in FCRL3 genes may affect the susceptibility to autoimmune diseases and exert their effects via different mechanisms. One possible consequence of FCRL3 polymorphisms is the variability in FCRL3 expression among patients via binding to NFkB transcription factor (18). Several types of immune cells such as B and T cells, express FCRL3. Therefore, aberrant activation of FCRL3 expression may result in biologically important alterations in immune parameters. Down-regulation of B-cell mediated signaling is probably one another mechanism which may help to breakdown of B-cell tolerance (19). Swainson et al. have also shown the impact of FCRL3 expression on the phenotype, differentiation, and function of T (reg). Their studies revealed the existence of FCRL3 (+) Treg subset characterized by lower responsiveness following antigenic stimulation and diminished suppressor activity (20).

In spite of the above findings, there also have been controversial reports regarding the relevance of the FCRL3 polymorphisms and predisposition to autoimmune disorders. Until now, there are no consensus results on the association of FCRL3 polymorphism and susceptibility to autoimmune disorders (21, 22). In addition, the association of the −169A/G SNP in FCRL3 of Iranian patients with BD observed in this study was not replicated in Chinese BD patients (14). The latter population showed a significantly higher frequency of the G allele at the −110 site in BD patients. Genetic heterogeneity, as a result of differences in allele frequencies is one possible explanation for this discrepancies. Another probable mechanism may be the existence of a linkage disequilibrium between this allele and neighboring unrecognized susceptibility genes.

This study has some limitations which need to be discussed. The first is ethnicity-dependent genetic risk factors for complex diseases such as BD. This genetic specificity provides variations in the rate of disease or disease onset age. Therefore, large-scale studies should be replicated across populations of different ethnicity. The outputs of such studies will clarify the association of these variants with susceptibility or resistance to disease. Another limitation is the absence of any experimental design for the assessment of gene-gene or gene-environment interactions. These biological interactions can deeply affect disease susceptibility and their evaluation can provide new insights into the genetic basis of disease.

## Conclusion

This study has revealed an association between predisposition to BD and FCRL3 gene polymorphisms. The observed allele and genotype frequencies of rs7528684 propose again a probable role for genetic influences on the development of autoimmunity and disease pathogenesis. Nonetheless, more studies are needed to further elucidate the molecular mechanisms underlying the interactions between environmental factors and genetic background in BD.

## Ethical considerations

Ethical issues (Including plagiarism, informed consent, misconduct, data fabrication and/or falsification, double publication and/or submission, redundancy, etc.) have been completely observed by the authors.
